# Antipsychotic use among paediatric patients living with epilepsy in Uganda

**DOI:** 10.1038/s41598-026-52732-z

**Published:** 2026-05-18

**Authors:** Joan Abaatyo, Anita Arinda, Henry Kawesi, Mark Mohan Kaggwa

**Affiliations:** 1 Department of Psychiatry, King Ceasor University, Kampala, Uganda; 2 Department of Psychiatry, Mulago Hospital, Kampala, Uganda; 3 Department of Psychiatry, School of Medicine, College of Health Sciences, Makerere University, Kampala, Uganda; 4 School of Medicine, College of Health Sciences, Makerere University, Kampala, Uganda; 5 Waypoint Centre for Mental Health Care, 500 Church Street, Penetanguishene, ON L9M 1G3 Canada; 6 Department of Psychiatry, University of Toronto, 250 College Street, 8Th Floor, Toronto, ON M5T 1R8 Canada

**Keywords:** Epilepsy, Children, Adolescents, Antipsychotics, Risperidone, Uganda, Diseases, Health care, Medical research, Neurology, Neuroscience

## Abstract

Epilepsy is a common chronic neurological disorder in childhood, often accompanied by behavioural and psychiatric challenges such as aggression, irritability, and hyperactivity. Management of these comorbidities may involve antipsychotic medications, yet data on their use in African settings remain scarce. To examine the prevalence and patterns of antipsychotic prescribing among children and adolescents with epilepsy in Uganda. A descriptive cross-sectional study was conducted at Mulago National Referral Hospital and Butabika National Mental Referral Hospital. Children (5–9 years) and adolescents (10–17 years) with epilepsy were consecutively recruited during routine visits. Data were obtained through caregiver interviews and review of medical records. Intellectual disability was evaluated with Raven’s Progressive Matrices. Antipsychotic and anti-seizure prescriptions were recorded. Data were analysed using descriptive statistics and chi-square tests. A total of 305 participants were recruited, mostly male (62.6%) and adolescents (51.8%). Overall, 6.9% (n = 21, 95% CI: 4.5–10.3) were prescribed antipsychotics, with risperidone being the most common (47.6%). The most common combinations involved carbamazepine with risperidone (38.1%) and sodium valproate with risperidone (28.6%). A statistically significant site-related difference was observed: nearly all individuals receiving antipsychotics were recruited from Mulago National Referral Hospital (9.1%), compared to only 1.2% from Butabika National Mental Referral Hospital (p = 0.014). Antipsychotic use among children and adolescents with epilepsy in Uganda is relatively low but follows international trends, with risperidone being the predominant choice. Antipsychotic prescriptions often involve combinations with carbamazepine or valproate, raising concerns about drug interactions and metabolic risks. These findings underscore the need for locally adapted prescribing guidelines, systematic monitoring of safety outcomes, and strengthened psychosocial interventions to complement pharmacological care.

## Introduction

Epilepsy is among the most common chronic neurological disorders in childhood, affecting an estimated 10.5 million children worldwide ^1–3^. The burden is particularly heavy in sub-Saharan Africa, where access to specialized neurological and mental health care is limited and treatment gaps remain substantial ^4^. Beyond recurrent seizures, many children and adolescents with epilepsy also experience behavioural and psychiatric challenges such as irritability, aggression, hyperactivity, anxiety, and mood disturbances ^5–8^. These comorbid difficulties can significantly impair quality of life, heighten caregiver stress, and complicate the overall management of epilepsy ^9^.

Management of behavioural challenges may include psychotropic medications, including antipsychotics, particularly in cases of severe aggression or comorbid neuropsychiatric conditions. ^10,11^. However, their use is typically reserved for selected cases after optimisation of seizure control and implementation of psychosocial interventions and is often off-label ^12,13^. Second-generation agents, including risperidone, olanzapine, and quetiapine, are commonly prescribed because of their relatively favourable safety and efficacy profile in paediatric populations ^12^. Nevertheless, concerns persist regarding their metabolic, neurological, and endocrine side effects, as well as their potential interactions with anti-seizure medications. In low-resource settings like Uganda, the use of antipsychotics for behavioural disturbances in epilepsy, often outside established indications, is frequently shaped by contextual constraints rather than standard guidelines ^12,14,15^. In these settings, treatment decisions are influenced not only by clinical indications but also by the limited availability of psychosocial interventions (e.g., behavioural therapy and occupational therapy), medication accessibility and affordability, and variability in clinician training and practice patterns ^16^. Additionally, drug–drug interactions between antipsychotics and anti-seizure medications (ASMs) are clinically significant ^17^. Enzyme-inducing ASMs such as carbamazepine can reduce plasma levels of antipsychotics, potentially reducing efficacy ^18,19^. These interactions occur globally but may be more impactful in low-middle income countries (LMICs) where therapeutic drug monitoring is rarely available ^20^.

Despite the clinical importance of this issue, little is known about antipsychotic use in children and adolescents with epilepsy in sub-Saharan Africa. Most existing evidence comes from high-income countries, which report the prevalence and patterns of psychotropic use in paediatric epilepsy and neurological populations ^10,14,21^. However, data from African contexts where health systems are constrained and clinical practices may vary across different levels of care remain scarce. This study sought to examine the use of antipsychotics among children and adolescents with epilepsy in Uganda.

## Methodology

### Study design and setting

This was a cross-sectional descriptive study conducted among children and adolescents with epilepsy at two national referral hospitals in Uganda: Mulago National Referral Hospital (MNRH) and Butabika National Mental Referral Hospital (BNMRH). These hospitals serve as major tertiary care facilities located in Kampala, the capital city of Uganda, offering both neurological and psychiatric services to patients nationwide.

### Study population and eligibility criteria

The study included children aged 5–9 years and adolescents aged 10–17 years with a confirmed diagnosis of epilepsy. aAge categories were defined using World Health Organization adolescent age classification  (10–19 years), with participants aged 10-17 years categorized as adolescents ^20^. Epilepsy diagnoses were made by qualified clinicians (neurologists, paediatricians, psychiatrists, psychiatry residents and paediatrics residents or trained clinical officers) based on clinical assessment and history.

Children under 5 years were excluded from this study for several reasons. First, the diagnosis of epilepsy in early childhood can be challenging, as seizure-like events in this age group may include a range of non-epileptic paroxysmal disorders (e.g., breath-holding spells, febrile seizures, and movement disorders), increasing the risk of misclassification ^22^. Additionally, epilepsy syndromes in early childhood are often heterogeneous and evolving, which may limit diagnostic certainty and comparability across participants ^22,23^. Second, prescribing practices in this age group differ substantially from those in older children and adolescents ^24^. The use of antipsychotic medications in children under 5 years is relatively uncommon and typically approached with greater caution due to limited evidence on safety, tolerability, and long-term neurodevelopmental effects ^25^. Dosing considerations, monitoring requirements, and regulatory approvals also differ in this population. Finally, behavioural manifestations in younger children may reflect developmental variation rather than distinct psychiatric comorbidity, making the interpretation of behavioural disturbances and their management less comparable to older age groups. For these reasons, restricting the study population to children aged 5 years and above allowed for greater diagnostic clarity and clinical homogeneity.

Participants were included if:

- They had a confirmed diagnosis of epilepsy
- They were on anti-seizure medication
- They had ongoing follow-up at either study site
- A primary caregiver was available to provide reliable information

Children with co-existing neurodevelopmental conditions were included, as these reflect real-world clinical populations.

Participants were recruited consecutively during routine outpatient visits and inpatient reviews between August 2024 and May 2025. Consecutive sampling was pragmatically employed to enhance representativeness within the study period.

### Data collection procedures

Data were collected through structured caregiver interviews and review of medical records. Caregivers provided information on socio-demographic characteristics, developmental history (including birth weight, speech and walking milestones), seizure history, and medication use. At Butabika, a diagnosis of epilepsy, as well as prescription of antipsychotics and anti-seizure medications, are made by psychiatrists, psychiatry residents, and psychiatric clinical officers. At Mulago, these responsibilities are shared between psychiatrists, psychiatry residents, psychiatric clinical officers, pediatricians, pediatric residents, and neurologists.

Intellectual ability/disability was assessed with Raven’s Progressive Matrices ^26,27^, a non-verbal measure of reasoning interpreted against age-appropriate norms. Evidence from Sub-Saharan Africa indicates that Raven’s tests show acceptable reliability and predictive validity in several African samples ^28^ and have been standardized locally in some contexts (e.g., Kenya) ^29^. However, convergent validity can vary, and norms may shift across cultural points we considered when interpreting scores ^30^.

Information on the prescription of antipsychotics and anti-seizure medications was obtained through review of patient files and medication charts, with all medications and combinations carefully documented.

### Study variables

The primary outcome variable was antipsychotic prescription (yes/no). Independent variables included socio-demographic, developmental, and clinical characteristics. Socio-demographic variables comprised age group (children 5–9 years, adolescents 10–17 years), sex (male, female), study site, and schooling status (school-going, not school-going).

Developmental variables included history of low birth weight (yes, no), speech delay (yes, no), current ability to speak (yes, no), delayed walking (yes, no), and current ability to walk (yes, no). Clinical variables captured epilepsy-related characteristics: age at first seizure (< 2 years vs ≥ 2 years), intellectual disability (yes, no), treatment adherence (suboptimal vs adherent), duration on anti-seizure medication (≤ 5 years vs > 5 years), and anti-seizure polypharmacy (monotherapy vs polytherapy).

Intellectual disability was assessed using Raven’s Progressive Matrices ^31^; however, severity could not be reliably categorized due to the lack of standardized local cut-offs.

ASM adherence was assessed by caregiver report using a structured question on missed doses: “How many doses of the child’s anti-seizure medication were missed in the past one month?” Responses were used to categorize adherence, with participants who had missed one or more doses classified as having suboptimal adherence, while those with no missed doses were classified as adherent.

### Ethical considerations

This study was conducted in accordance with the ethical principles of the Declaration of Helsinki and all applicable local regulations ^32^. Ethical approval for the larger study from which this data was drawn was obtained from the Makerere University School of Medicine Research Ethics Committee (Ref. Mak-SOMREC-2024–889) and the Uganda National Council of Science and Technology (Ref. HS4589ES). Written informed consent was obtained from caregivers, and assent was sought from children and adolescents aged 8 years and above. Participation was voluntary, and confidentiality was maintained through the anonymized collection and secure storage of data.

### Data analysis

Data analysis was conducted using STATA version 17.0. Categorical variables were summarised as frequencies and percentages. Chi-square tests were used to assess associations.

## Results

A total of 305 participants, most of whom were adolescents (51.8%) and males (62.6%) were included in the study. The majority were from Mulago National Referral Hospital (72.1%), and 61.6% were school going. A history of low birth weight was reported in 10.0%. Developmental delays were common, with 37.0% experiencing delayed speech and 33.9% experiencing delayed walking; however, most were able to speak (75.4%) and walk (85.9%) at the time of assessment. About one-third (32.5%) were on more than one ASM, and 35.7% had intellectual disability. See Table 1

**Table 1:** Cross-tabulation of the characteristics by antipsychotic prescription status.

Variable	n (%)	Antipsychotic		X^2^ (p-value)
		No 284 (93.1)	Yes 21 (6.9)	
Age-group				
Children (5 – 9 years)	147 (48.2)	135 (91.8)	12 (8.2)	0.72 (0.395)
Adolescents (10 – 17 years)	158 (51.8)	149 (94.3)	9 (5.7)	
Sex				
Female	114 (37.4)	108 (94.7)	6 (5.3)	0.75 (0.387)
Male	191 (62.6)	176 (92.1)	15 (7.9)	
Study site				
BNMRH	85 (27.9)	84 (98.9)	1 (1.2)	**5.99 (0.014)**
MNRH	220 (72.1)	200 (90.1)	20 (9.1)	
School-going				
No	117 (38.4)	107 (91.5)	10 (8.5)	0.82 (0.366)
Yes	188 (61.6)	177 (94.1)	11 (5.9)	
Had low birth weight				
No	215 (90.0)	201 (93.5)	14 (6.5)	0.11 (0.735)
Yes	24 (10.0)	22 (91.7)	2 (8.3)	
Experienced speech delay				
No	133 (63.0)	121 (91.0)	12 (9.0)	1.06 (0.303)
Yes	78 (37.0)	74 (94.1)	4 (5.1)	
Currently able to speak				
No	75 (24.6)	71 (94.7)	4 (5.3)	0.37 (0.541)
Yes	230 (75.4)	213 (92.6)	17 (7.4)	
Experienced delayed walking				
No	160 (66.1)	146 (91.2)	14 (8.8)	0.53 (0.468)
Yes	82 (33.9)	77 (93.9)	5 (6.1)	
Currently able to walk				
No	43 (14.1)	42 (97.7)	1 (2.3)	1.63 (0.203)
Yes	262 (85.9)	242 (92.4)	20 (7.6)	
Had first seizure at > / = 2 years				
No	166 (56.9)	154 (92.8)	12 (7.2)	0.09 (0.768)
Yes	126 (43.1)	118 (93.7)	8 (6.3)	
Anti-seizure polypharmacy				
No	206 (67.5)	190 (92.2)	16 (7.8)	0.77 (0.380)
Yes	99 (32.5)	94 (95.0)	5 (5.0)	
Has Intellectual disability				
No	196 (64.3)	186 (94.9)	10 (5.1)	2.72 (0.099)
Yes	109 (35.7)	98 (89.9)	11 (10.1)	
Treatment adherence				
Sub-optimal	260 (85.8)	241 (92.7)	19 (7.3)	0.40 (0.525)
Adherent	43 (14.2)	41 (95.3)	2 (4.7)	
Duration on anti-seizure medication treatment				
< / = 5 years	236 (78.7)	219 (92.8)	17 (7.2)	0.07 (0.791)
> 5 years	64 (21.3)	60 (93.7)	4 (6.3)	

### Prevalence of antipsychotic use

Antipsychotic use was observed in 6.9% of participants (n = 21; 95% Confidence Interval [CI]: 4.5–10.3%). A significant site-related difference was noted, with more individuals recruited from Mulago National Referral Hospital (9.1%) being prescribed more antipsychotics compared to those from Butabika National Mental Referral Hospital 1.2% (p = 0.014). (Table 1).

### Distribution of antipsychotics and ASM combinations among children and adolescents with epilepsy

Among those on antipsychotics, the most frequently prescribed antipsychotic was Risperidone (n = 10, 47.6%). The most frequently prescribed combination was carbamazepine with risperidone (38.1%), followed by sodium valproate with risperidone (28.6%). See Table 2

**Table 2:** Antipsychotic and Anticonvulsant Combinations Prescribed Among Children and Adolescents with Epilepsy and Behavioral Challenges (n = 21).

Combination of medications	Frequency (n)	Percentage (%)
Carbamazepine + Stelazine	1	4.8
Carbamazepine + Risperidone	8	38.1
Carbamazepine + Haloperidol	2	9.5
Carbamazepine + Levetiracetam + Risperidone	1	4.8
Sodium Valproate + Risperidone	6	28.6
Sodium Valproate + Carbamazepine + Risperidone	1	4.8
Sodium Valproate + Carbamazepine + Haloperidol	1	4.8
Sodium Valproate + Carbamazepine + Olanzapine	1	4.8

## Discussion

This study addresses this knowledge gap by examining the use of antipsychotics among children and adolescents with epilepsy in multiple hospitals in Uganda. This study found that risperidone was the most prescribed antipsychotic for children and adolescents with epilepsy in Uganda, often combined with carbamazepine or valproate, raising concerns about drug interactions and metabolic risks.

The higher rate of antipsychotic prescribing observed at Mulago compared to Butabika may reflect differences in facility type and clinical staffing. While Mulago is a general hospital with a broader mix of clinicians ^33^, Butabika is a specialised mental health facility and may have greater access to multidisciplinary services, including occupational therapy and other behavioural interventions ^34^. Greater availability of non-pharmacological approaches may reduce reliance on antipsychotic medications for behavioural management. However, these factors were not directly assessed in this study and should be interpreted with caution.

The observation that risperidone was the most prescribed antipsychotic, consistent with broader paediatric prescribing patterns ^21^. However, guideline-supported indications are largely restricted to irritability and aggression in autism spectrum disorder and, to a lesser extent, intellectual disability ^10,11^. As the specific rationale for antipsychotic prescription and co-existing conditions were not captured, it was not possible to determine whether use was guideline-concordant. Its frequent use alongside carbamazepine and sodium valproate reflects common practice, combining first-line ASMs with second-generation antipsychotics like risperidone ^10,14,21^. However, this combination raises concerns: carbamazepine can reduce risperidone plasma concentrations through enzyme induction, potentially diminishing its effectiveness ^35,36^. While alternative agents such as olanzapine or quetiapine may be considered in such contexts ^10^, this study did not evaluate comparative effectiveness or safety. The predominance of risperidone in our sample may instead reflect contextual factors, including availability, affordability, and clinician familiarity, rather than a demonstrated therapeutic advantage ^37,38^. These findings highlight the need for context-specific research to guide optimal antipsychotic selection in paediatric epilepsy.

The second most common combination of valproate and risperidone raises the risk of weight gain and metabolic syndrome ^39,40^. An RCT found that combining risperidone with divalproex led to a 3.4 kg weight increase over 28 days ^41^. Nonetheless, concerns increase with a case report noting a 90 kg weight gain in a patient on this combination within seven months ^42^. These findings highlight the importance of monitoring and managing metabolic effects, an aspect that may be difficult to implement in a resource-limited setting like Uganda ^43^. However, this study did not collect metabolic outcome data due to its unavailability in records, highlighting an important gap in routine monitoring.

The frequent use of risperidone alongside carbamazepine or valproate may reflect a pragmatic approach to managing behavioural comorbidities in epilepsy, where symptoms can stem from the disorder itself or from psychiatric and behavioural side effects of ASMs (such as irritability and mood changes), leading to the use of antipsychotics as adjuncts ^44^. Our results align with international trends that favour risperidone for aggression and irritability in young people but also highlight specific risks in epilepsy management. These include pharmacokinetic interactions with enzyme-inducing ASMs and increased metabolic burden, issues that are less often emphasized in studies outside epilepsy populations. These findings advocate for context-specific guidelines and encourage prospective studies to evaluate the effectiveness, tolerability, and long-term outcomes of ASM–antipsychotic combinations in paediatric epilepsy services ^45^.

### Strengths and limitations

This study has several strengths. Firstly, it is among the few multi-centre studies from sub-Saharan Africa that investigate antipsychotic use in children with epilepsy, providing valuable data from a low-resource setting where evidence is limited. Additionally, data collection combined caregiver interviews with medical record reviews, enhancing the completeness and reliability of treatment information.

Despite these strengths, several limitations should be acknowledged. The cross-sectional design limits causal inference, and reliance on caregiver reports and medical records may have introduced recall bias and incomplete data. Anti-seizure medication adherence was assessed via caregiver report, which may be subject to recall and social desirability bias. While children with comorbid neurological or neurodevelopmental conditions were included, these conditions were not comprehensively assessed or individually characterised. Although intellectual disability was included in the analysis, other co-existing conditions (e.g., autism spectrum disorder, or cerebral palsy) and the specific clinical rationale for antipsychotic prescription were not captured. This limits our ability to determine whether prescribing was guideline-concordant or attributable specifically to epilepsy-related behavioural difficulties. Additionally, because the sample was taken from two tertiary referral hospitals, the findings might not reflect prescribing practices at lower-level health facilities or rural areas. The study did not evaluate treatment outcomes, adverse effects, or long-term safety, leaving questions about the risk–benefit ratio. Furthermore, the absence of routinely documented metabolic or anthropometric data limited our ability to assess monitoring practices for antipsychotic-related adverse effects.

## Conclusion

Antipsychotic prescribing for children and adolescents with epilepsy in Uganda follows global trends but is shaped by local realities. Risperidone is the most prescribed agent, often combined with carbamazepine or sodium valproate, raising concerns about drug interactions and metabolic risks. These patterns reflect both clinical need and systemic constraints, including limited access to non-pharmacological care. The findings highlight the urgent need for locally adapted guidelines, regular monitoring of safety outcomes, and expanded psychosocial interventions to ensure safe, equitable, and effective management of behavioural comorbidities in paediatric epilepsy.

## Data Availability

Data will be made available upon reasonable request to the corresponding author.

## Declarations

### Clinical trial registration

This study is not a clinical trial.
